# Retraction: Mesenteric Panniculitis: A Rare Condition in a Patient With Rheumatoid Arthritis

**DOI:** 10.7759/cureus.r108

**Published:** 2024-01-25

**Authors:** Thamer S Alzahrani, Ali H Alharbi, Abdulellah I Al Homoudi, Arwa A Reidi, Farah A Alshehri, Nawaf F Alsarraj, Heba H Milibary, Rashed A Alnemer, Yousef Z Murad, Sarah M Almousa, Bashaier M Albalawi, Ayman J Almalky, Ali B Alaithan, Furqan H Alawami, Faisal Al-Hawaj

**Affiliations:** 1 College of Medicine, Al-Baha University, Al-Baha, SAU; 2 General Practice, Al-Nakheel Primary Health Care Center, Qassim, SAU; 3 College of Medicine, Dar Al Uloom University, Riyadh, SAU; 4 College of Medicine, Ibn Sina National College for Medical Studies, Jeddah, SAU; 5 College of Medicine, King Abdulaziz University, Jeddah, SAU; 6 College of Medicine, King Saud Bin Abdulaziz University for Health Sciences, Riyadh, SAU; 7 College of Medicine, Tabuk University, Tabuk, SAU; 8 College of Medicine, Taif University, Taif, SAU; 9 College of Medicine, Vision Colleges, Riyadh, SAU; 10 College of Medicine, Imam Abdulrahman Bin Faisal University, Dammam, SAU

The Editors-in-Chief have retracted this article. Concerns were raised regarding the identity of the authors on this article. Specifically, Faisal Alhaway and Malak Shammari have stated that they were added to this article without their knowledge or approval. The identity of the other authors could also not be verified. As the appropriate authorship of this work cannot be established, the Editors-in-Chief no longer have confidence in the results and conclusions of this article.

